# Identification and Partial Characterization of Potential FtsL and FtsQ Homologs of *Chlamydia*

**DOI:** 10.3389/fmicb.2015.01264

**Published:** 2015-11-13

**Authors:** Scot P. Ouellette, Kelsey J. Rueden, Yasser M. AbdelRahman, John V. Cox, Robert J. Belland

**Affiliations:** ^1^Division of Basic Biomedical Sciences, Sanford School of Medicine, University of South DakotaVermillion, SD, USA; ^2^Department of Microbiology, Immunology, and Biochemistry, University of Tennessee Health Science CenterMemphis, TN, USA; ^3^Department of Microbiology and Immunology, Faculty of Pharmacy, Cairo UniversityCairo, Egypt

**Keywords:** *Chlamydia*, cell division, ftsQ, ftsL, bacterial two-hybrid system

## Abstract

*Chlamydia* is amongst the rare bacteria that lack the critical cell division protein FtsZ. By annotation, *Chlamydia* also lacks several other essential cell division proteins including the FtsLBQ complex that links the early (e.g., FtsZ) and late (e.g., FtsI/Pbp3) components of the division machinery. Here, we report chlamydial FtsL and FtsQ homologs. Ct271 aligned well with *Escherichia coli* FtsL and shared sequence homology with it, including a predicted leucine-zipper like motif. Based on *in silico* modeling, we show that Ct764 has structural homology to FtsQ in spite of little sequence similarity. Importantly, *ct271/ftsL* and *ct764/ftsQ* are present within all sequenced chlamydial genomes and are expressed during the replicative phase of the chlamydial developmental cycle, two key characteristics for a chlamydial cell division gene. GFP-Ct764 localized to the division septum of dividing transformed chlamydiae, and, importantly, over-expression inhibited chlamydial development. Using a bacterial two-hybrid approach, we show that Ct764 interacted with other components of the chlamydial division apparatus. However, Ct764 was not capable of complementing an *E. coli* FtsQ depletion strain in spite of its ability to interact with many of the same division proteins as *E. coli* FtsQ, suggesting that chlamydial FtsQ may function differently. We previously proposed that *Chlamydia* uses MreB and other rod-shape determining proteins as an alternative system for organizing the division site and its apparatus. Chlamydial FtsL and FtsQ homologs expand the number of identified chlamydial cell division proteins and suggest that *Chlamydia* has likely kept the late components of the division machinery while substituting the Mre system for the early components.

## Introduction

**FIGURE 1:**
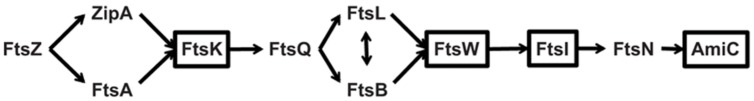
**A bacterial cell division pathway based on *Escherichia coli*.** The cell division machinery is recruited and organized in an hierarchical and temporal manner beginning with the recruitment of FtsZ to the division site and ending with the separation of daughter cells by the amidases. The boxed proteins are those for which *Chlamydia* has an annotated homolog.

Canonical bacterial cell division begins with the recruitment of the bacterial GTPase tubulin homolog FtsZ to the division plane (Bi and Lutkenhaus, 1991; de Boer et al., 1992; Mukherjee et al., 1993). Subsequently, division proteins are recruited in a temporal and hierarchical manner to effect the synthesis of a peptidoglycan cell wall at the division site (Begg and Donachie, 1985). FtsZ and the Z-ring complex (e.g., FtsA, ZipA) are the first components recruited to the division plane, after which there is a temporal delay in the recruitment of the peptidoglycan synthesis protein complex (e.g., FtsI, FtsW; Aarsman et al., 2005). A key set of proteins in this process is the FtsLBQ complex (Buddelmeijer and Beckwith, 2004), which links the early Z-ring complex to the late peptidoglycan complex (see **Figure 1**). FtsLBQ is thought to provide a structural scaffold for these interactions (Villanelo et al., 2011) with recent evidence suggesting FtsL can trigger the actual division process (Tsang and Bernhardt, 2015). Not surprisingly, most of these proteins and other conserved Fts division proteins are essential in many bacteria.

*Chlamydia* is a Gram-negative, obligate intracellular bacterial pathogen that causes a range of illnesses in humans and animals (Schachter et al., 1973). It alternates between functionally and morphologically distinct forms during its developmental cycle. The EB, or elementary body, mediates attachment and internalization into a susceptible host cell whereas the RB, or reticulate body, grows and divides within a membrane bound compartment termed an inclusion. In evolving to obligate intracellular parasitism, *Chlamydia* has streamlined its genome by eliminating superfluous pathways and genes. Conversely, if *Chlamydia* has maintained a set of genes, then it is likely important for the bacterium. Surprisingly, amongst the genes *Chlamydia* has deleted are several *fts* cell division genes including *ftsZ* (Stephens et al., 1998). Conversely, *Chlamydia* has maintained several rod-shape determining genes, including the bacterial actin homolog *mreB*, in spite of being a coccoid bacterium (Ouellette et al., 2012). We previously identified and presented evidence that *Chlamydia* uses rod-shape determining proteins for cell division (Ouellette et al., 2012, 2014a). We further hypothesized that *Chlamydia* has substituted MreB for FtsZ in the division process (Ouellette et al., 2012), and another lab subsequently showed localization data for MreB at chlamydial division planes (Kemege et al., 2015). Taking these rod-shape determining genes into account, the annotated chlamydial genome still lacks several of the later division components such as *ftsLBQN*. Thus, how *Chlamydia* divides without FtsZ and other critical divisome components remains an intriguing question.

We hypothesized that *Chlamydia* encodes a full complement of “late” division genes, given the presence of *ftsK*, *ftsW*, and *ftsI* that were identified based on sequence similarity in the original annotation of the *Chlamydia trachomatis* genome sequence (**Figure 1**; Stephens et al., 1998). We initiated a systematic search to find the “missing” genes. In doing so, we found that the hypothetical chlamydial protein, Ct764, displays *in silico* structural homology to FtsQ. We also noted that the gene immediately upstream from *ftsI* in *Chlamydia*, *ct271*, aligns well with the gene immediately upstream of *ftsI* in *Escherichia coli*, *ftsL*. Searching for Ct271 and Ct764 orthologs in other chlamydial organisms revealed the presence of such orthologs in all sequenced genomes-an important consideration for a chlamydial division protein. The transcription of both genes was consistent with the replicative (RB) phase of the chlamydial developmental cycle, a time when division occurs. GFP-Ct764 localized to chlamydial division septa, and, when over-expressed, blocked chlamydial development. One characteristic of cell division proteins is that they interact with each other, and this can be exploited in searching for them. Using a bacterial two-hybrid approach, Ct764 was shown to interact with known chlamydial division proteins as well as division proteins from *E. coli*. However, Ct764 did not complement an *E. coli ftsQ* depletion strain. Taken together, our data indicate that Ct271 and Ct764 are chlamydial FtsL and FtsQ homologs, respectively.

## Materials and Methods

### Organisms and Cell Culture

*Chlamydia trachomatis* serovar L2 EBs were harvested from infected cell cultures at 37°C with 5% CO_2_, purified by discontinuous density gradient centrifugation in Renografin (Bracco Diagnostics, Princeton, NJ, USA), and titred for infectivity by determining inclusion forming units (IFU) on fresh cell monolayers. *C. trachomatis* serovar L2 lacking the endogenous plasmid (-pL2) was a kind gift of Dr. I. Clarke. McCoy cells were routinely cultivated at 37°C with 5% CO_2_ in DMEM containing glutamine, glucose, pyridoxal, and sodium bicarbonate (Sigma, St. Louis, MO, USA) and further supplemented with 10% FBS. HEp-2 cells were routinely cultivated at 37°C with 5% CO_2_ in IMDM containing glutamax, glucose, HEPES, and sodium bicarbonate (Gibco) supplemented with 10% FBS. The McCoy and HEp-2 cells were kind gifts of Dr. H. Caldwell (Rocky Mountain Labs/NIAID). The adenylate cyclase mutant strain (Δ*cya*) DHT1 (Dautin et al., 2000) and XL1 strain (Stratagene, Santa Clara, CA, USA) of *E. coli* were cultured in LB broth. Chemically competent cells of all *E. coli* were prepared following standard protocols. All antibiotics and chemicals used in the described experiments were purchased from Sigma (St. Louis, MO, USA) unless otherwise indicated. IPTG, X-gal, and all restriction and other DNA-modifying enzymes were obtained from Fermentas (Thermo Fisher, Rockford, IL, USA) whereas the components for the Gateway^^®^^ system were obtained from Invitrogen.

### Bioinformatics Analyses

**FIGURE 2:**
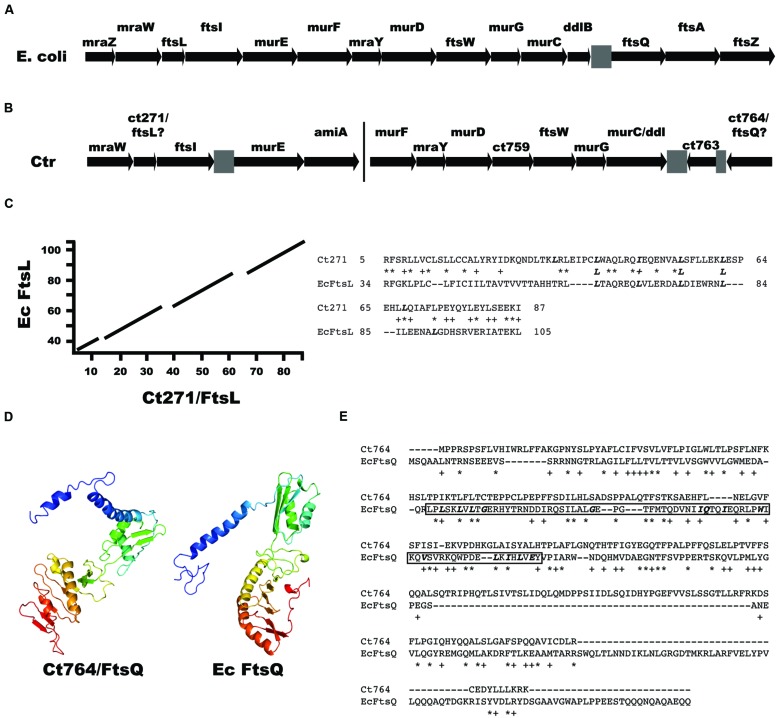
**Identification of *ftsL* and *ftsQ* homologs in *Chlamydia*. (A)** Arrangement of the *dcw* gene cluster from *E. coli* encoding cell division and peptidoglycan synthesis genes. Note the presence of *ftsL* upstream of *ftsI* and *ftsQ* downstream of *ddlB*. **(B)** Arrangement of cell division and peptidoglycan synthesis genes in *Chlamydia*. The bar indicates that the clusters are separated on the chromosome. Note the presence of *ct271* upstream of *ftsI*. For **(A)** and **(B)**, gray boxes are intergenic regions. **(C)** A BLAST alignment of *E. coli* FtsL (EcFtsL; 121 amino acids) vs. chlamydial Ct271 (95 amino acids) and the corresponding sequence alignment of the indicated amino acids. Bolded, italicized Leu/Iso residues indicate the leucine zipper-like motifs within the FtsL homologs. **(D)** Phyre2 structural prediction of Ct764 and the structure of *E. coli* FtsQ. The Ct764 model had a 98.8% confidence match to *E. coli* FtsQ. Ribbon diagrams are color-coded red to violet (Nter to Cter). **(E)** Clustal Omega alignment of Ct764 and *E. coli* FtsQ (EcFtsQ). Key residues in the POTRA domain of *E. coli* FtsQ are bolded and italicized with the domain itself boxed (amino acids 55–126). For **(C)** and **(E)**, ^∗^ = identical residues; + = similar residues.

Gene maps and sequences for *C. trachomatis* serovar D were obtained from the STD sequence database^1^ and for *E. coli* from the Ecogene 3.0 project^2^. Alignments were performed using the NCBI Nucleotide BLAST function (**Figure 2C**; Altschul et al., 1990). Structural homology was performed using the Phyre 2 structural prediction server and the “intensive” search function (**Figure 2D**; Kelley et al., 2015^3^). Multiple sequence alignments and gene clustering analyses were performed using the EMBL Clustal Omega server using the default settings (**Figure 2E**; Sievers et al., 2011^4^). Transmembrane domains were predicted using the TOPCONS (Tsirigos et al., 2015^5^) and TMPred servers (Hofmann and Stoffel, 1993^6^). Proteins sequences were analyzed for signal sequences using the SignalP 4.1 server (Petersen et al., 2011^7^). Leucine zipper-like motifs were detected using 2Zip server (Bornberg-Bauer et al., 1998^8^).

### RT-qPCR

For transcript analyses, HEp-2 cells were plated in 6-well plates at a density of 10^6^ per well and infected at an MOI of 1 with *C. trachomatis* serovar L2. Assays to quantify the indicated transcripts were performed as described previously (Ouellette et al., 2005, 2006). Briefly, total RNA was collected from infected cells at the indicated times using Trizol (Invitrogen/Thermo) and treated with Turbo DNAfree [Ambion (Life Technologies)] to remove contaminating DNA, according to the manufacturer’s guidelines. 1 μg DNA-free RNA was reverse-transcribed with random nonamers (New England Biolabs, Ipswich, MA, USA) using SuperScript III RT (Invitrogen/Thermo) according to the manufacturer’s instructions. Equal volumes of cDNA were used in qPCR reactions with SYBR Green (Quanta Biosciences, Gaithersburg, MD, USA) and measured on an ABI 7300 system [Applied Biosystems (Life Technologies)]. Duplicate DNA samples were collected from the same experiment using DNeasy Tissue kit (Qiagen). Chlamydial genomes were quantified from equal amounts of total DNA by qPCR as above and used to normalize transcript data as described (Ouellette et al., 2005, 2006). Transcript levels of *mreB, rodZ, euo*, and *omcB* were determined using previously described primer sets (Ouellette et al., 2014a). For *ct271/ftsL*, the forward primer 5′-TCCCTTGTTTATGGGCTCAAT and the reverse primer 5′-CAACAAATGTTCAGGGCTTTCT were used. For *ct764/ftsQ*, the forward primer 5′-GCACTCCCTAACGCCTATTAAG and the reverse primer 5′-GGAATCAGCAGAAAGATGCAAG were used. For *ct739/ftsK*, the forward primer 5′-CGACTCCAAGTTCCTCTTCTTC and the reverse primer 5′-GATCCAGTGGTTCCTGCAATA were used.

### Chlamydial Transformation and Quantification of Inclusion Forming Units (IFU)

*Chlamydia trachomatis* serovar L2 lacking the endogenous plasmid (-pL2) was transformed with the tetracycline-inducible plasmids described in Supplemental Table S1 following published protocols (Wang et al., 2011; Wickstrum et al., 2013). Transformants were subsequently plaque purified as described elsewhere (Matsumoto et al., 1998). To determine GFP-Ct764 localization in chlamydiae, HEp-2 cells were plated on coverslips in 24-well plates at a density of 7 × 10^4^ per well and subsequently infected with the transformed strain in the presence of 2U/mL penicillin and 1 μg/mL cycloheximide. The infection was allowed to progress for 16–18 hpi before addition of 1 nM anhydrotetracycline (aTc). After 1–6 h of induction, cells were fixed in methanol for 5′ at room temperature and subsequently stained with a primary guinea pig antibody (kind gift of Dr. E. Rucks: University of South Dakota) and secondary goat anti-guinea pig DyLight405 (Jackson ImmunoResearch Labs, West Grove, PA, USA). Images were acquired using an Olympus FluoView 1000 laser scanning confocal microscope with a 60x objective (**Figure 4A**). For division septum localization of GFP-Ct764, infected cells were induced with 0.06 nM aTc at 9 hpi and fixed at 12 hpi in 3% formaldehyde/0.045% glutaraldehyde in PBS for 10 min followed by permeabilization with 90% methanol for 10 min. GFP-Ct764 was detected by staining with a mouse monoclonal anti-GFP antibody and chlamydial membranes with goat polyclonal anti-MOMP antibodies (Meridian Life Science) followed by donkey secondary AlexaFluor antibodies (594 and 488, respectively; Invitrogen). Images were acquired using a 100x Plan-Apochromat oil immersion lens on a Zeiss AxioImager.M2 equipped with a motorized stage and an Axiocam MRm camera. Images were deconvolved using Axiovision 4.7 software and processed with only linear adjustment of brightness and contrast and color palette switching to make the GFP stain green and the MOMP stain blue. To determine the effect of over-expression of GFP-Ct764, HEp-2 cells were plated in 6-well plates at a density of 10^6^ per well. In a subset of wells, cells were plated on coverslips. Approximately 18 h later, confluent cell monolayers were rinsed with PBS, and 10^6^ IFUs of wild-type *C. trachomatis* (L2), *C. trachomatis* L2 transformed with the empty pASK-gfp::L2 vector, or *C. trachomatis* L2 transformed with pASK-gfp_ct764::L2 were added directly to each well, and infected cells were incubated at 37°C with 5% CO_2_ in the presence (for transformants) or absence (wild-type) of 2 U/mL penicillin and 1 μg/mL cycloheximide. At 16 hpi, 10 nM aTc was added directly to the medium, and cells were incubated for an additional 24 h. Infected cells were rinsed with PBS, and 1 mL of SPG (sucrose/phosphate/glutamate) buffer was added to each well. Cells were scraped and collected from each well into a 1.5 mL microfuge tube with three glass beads. Samples were vortexed for 45 s and frozen at -80°C. Samples were titred for infectivity on fresh cell layers to quantify the number of IFU per well (Ouellette et al., 2006). Coverslips were fixed in methanol and processed for immunofluorescence as described above. Epifluorescent microscopy images were acquired using an Olympus BX60 with a 20x objective and a Nikon DS-Qi1MC digital camera.

### Cloning

A list of primers and plasmids used in these studies can be viewed in Supplemental Table S1. PCR was performed using Phusion DNA polymerase (Thermo Fisher) against purified genomic DNA from *C. trachomatis* serovar L2 or *E. coli* MG1665 following the manufacturer’s guidelines. PCR products were purified using the PCR purification kit (Qiagen, Valencia, CA, USA) and digested with the indicated restriction enzymes. Empty vector pBRtet57 plasmid was similarly digested and treated with alkaline phosphatase before ligation (with T4 DNA ligase) to digested PCR products. Chemically competent XL1 cells were transformed and plated on selective antibiotics in the presence of 0.4% glucose. Plasmids were prepared from colonies containing the correct sequence-verified construct. For Gateway^^®^^ constructs, full-length open reading frames for the genes of interest (lacking start and stop codons) cloned into pDONR221 were obtained from the Pathogen Functional Genomic Resource Center^9^. The ORF was transferred to the Gateway^^®^^-derivatized BACTH plasmids, pST25-DEST or pUT18C-DEST (Ouellette et al., 2014b), using the LR reaction (Invitrogen) following the manufacturer’s guidelines. *E. coli* BACTH expression constructs are described in Karimova et al. (2005, 2009).

### BACTH and β-galactosidase Assays

Bacterial adenylate cyclase two hybrid (BACTH) interactions and β-galactosidase measurements were performed as described elsewhere (Karimova et al., 2005; Gauliard et al., 2015) using the adenylate cyclase mutant (Δ*cya*) DHT1 strain of *E. coli*. Briefly, chemically competent DHT1 were co-transformed with each plasmid to be tested and plated on M63 minimal medium agar containing selective antibiotics, 40 μg/mL X-gal, 0.5 mM IPTG, and 0.2% maltose (Karimova et al., 1998). Plates were incubated at 30°C for up to 5 days for heterologous interactions between the Pbp constructs and those of *E. coli*. 8 colonies from each plate were cultured for 24 h at 30°C in 96-well plate format in 300 μL minimal medium broth containing selective antibiotics, IPTG, and maltose. These cultures were diluted to 1mL in minimal medium the following day. 200 μL was used for OD_600_ measurement and 200 μL was used to lyse the bacteria for the β-galactosidase measurement and subsequently incubated with 0.4% ONPG and visualized at OD_405_. β-galactosidase activity is expressed as 1000^∗^(OD_405_/OD_600_)/min after subtracting the blanks for medium alone.

### *Escherichia coli* FtsQ Depletion Strain and Complementation

The *ftsQ* depletion strain (JOE417) has been previously characterized (Chen et al., 2002). JOE417 was a kind gift of Dr. M. Goldberg (Harvard Medical School) and was routinely cultured in LB supplemented with kanamycin, chloramphenicol, and 0.2% arabinose. Chemically competent JOE417 were transformed with pBRtet57-ct764 (expressing Ct764), pBRtet57-Ec_ftsQ (expressing *E. coli* FtsQ), or the empty vector control, pBRtet57, and plated on Luria agar with the indicated supplements plus ampicillin for pBRtet57. For complementation experiments, overnight cultures of each transformant were diluted 1:100 in LB with ampicillin, kanamycin, and 0.2% arabinose for approximately 3 h. Cultures were normalized to OD_600_ = 1 and serial 1:10 dilutions were made in LB only. 5 μL of each dilution from 10^-1^ to 10^-6^ were spotted onto permissive agar medium containing kanamycin, chloramphenicol, ampicillin, and 0.2% arabinose or non-permissive medium containing kanamycin, ampicillin, 0.4% glucose, and varying concentrations of anhydrotetracycline from 0.2 to 10 nM. Plates were incubated overnight at 30°C and imaged using a Syngene Ingenius Bioimaging System (Synoptics Group, Cambridge, UK). Images were processed equally for brightness and contrast using Adobe Photoshop CS6 (San Jose, CA, USA).

## Results

### Identification of Chlamydial ftsL and ftsQ Homologs

We initiated a detailed analysis of all *C. trachomatis* hypothetical proteins in close proximity on the chromosome to known/annotated cell division genes, hypothesizing that such proteins may represent cell division components. For example, in *E. coli*, the DCW cluster encodes several peptidoglycan synthesis (*mur*) and cell division (*fts*) genes followed immediately by the co-directional *ftsQAZ* operon (Blattner et al., 1997; **Figure 2A**). In *Chlamydia*, a similar arrangement is seen with *mur* genes and *ftsW* (**Figure 2B**), although two different gene clusters exist on the chromosome. Indeed, the protein sequence of gene *ct271*, located upstream of *ftsI*, aligned with FtsL from *E. coli* (**Figure 2C**), the gene for which occupies the same chromosomal position relative to *ftsI*. Importantly, *ct271* orthologs are present in all sequenced chlamydial genomes in the same position relative to *ftsI* (data not shown). Chlamydial FtsL is 95 amino acids and encodes a putative transmembrane domain at the N-terminus of the protein (residues 6–24; TMpred) that is not predicted to be a signal sequence as determined by SignalP (Petersen et al., 2011). The alignment it shares with the 121 amino acid protein FtsL from *E. coli* spans the transmembrane and periplasmic domains, yet the sequence homology between the two proteins is not significant (*E*-value = 0.12), likely owing to their small sizes. However, the proteins do share 25% identity/40% similarity over 87% of Ct271. As a comparison, Ct270/FtsI (647 residues) shares 30% identity/45% similarity over 74% of its sequence when compared to *E. coli* FtsI (588 residues; Ouellette et al., 2012). Yet, the larger sequence allows for significance (*E*-value = 2e-46). FtsL contains a leucine zipper-like motif, with leucine residues every seven amino acids (italicized and bolded in **Figure 2C**), important for its interaction with FtsB (Buddelmeijer and Beckwith, 2004; Robichon et al., 2011). Ct271 also contains such a motif within its predicted periplasmic region (**Figure 2C**) albeit with one isoleucine residue substituted for one of the leucines. Thus, *Chlamydia* appears to encode a potential FtsL homolog.

Not surprisingly given the absence of FtsZ in chlamydial genomes, the downstream co-directional *ftsQAZ* operon appears to have been deleted in *Chlamydia*. Rather, the downstream genes of unknown function, *ct763* and *ct764*, are present in an inverted orientation. By running a series of bioinformatics analyses including BLAST alignments, transmembrane domain predictions (TOPCONS/TMpred), and *in silico* structural analyses, we identified the 268 amino acid protein Ct764 as a potential FtsQ homolog. Although Ct764 showed no BLAST sequence homology (data not shown), the protein was found to have structural homology to FtsQ homologs based on the Phyre2 structural prediction server (**Figure 2D**; confidence 98.8% compared to *E. coli* FtsQ). Further, Ct764 showed a similar, bitopic membrane protein domain architecture as FtsQ by possessing a short cytoplasmic tail, a single transmembrane domain from amino acids 27 to 48 (as predicted by TOPCONS; Tsirigos et al., 2015), and a large periplasmic domain. For comparison, the 276 amino acid protein FtsQ from *E. coli* encodes a transmembrane domain from amino acids 23 to 44 as predicted by TOPCONS. Interestingly, Ct764 has not conserved many of the amino acids comprising the POTRA domain that has been described for FtsQ (**Figure 2E**; see also Sánchez-Pulido et al., 2003), and no identifiable POTRA domain was identified in BLAST searches (data not shown). The POTRA domain is thought to play an important role in recruitment of FtsQ to the division septum (van den Ent et al., 2008). Finally, all sequenced genomes of *Chlamydia* and related species encode a Ct764 ortholog (Supplemental Figure S1), which is an important consideration for a chlamydial cell division protein. From these data, we hypothesized that Ct764 is a chlamydial FtsQ homolog. It thus appears that *Chlamydia* inverted the orientation of the *ftsQAZ* operon and deleted *ftsAZ* since Ct763 has a predicted role in RNA metabolism (Stephens et al., 1998) and *Chlamydia* lacks FtsA and FtsZ homologs.

### ct271 and ct764 are Transcribed during RB Growth and Division

**FIGURE 3:**
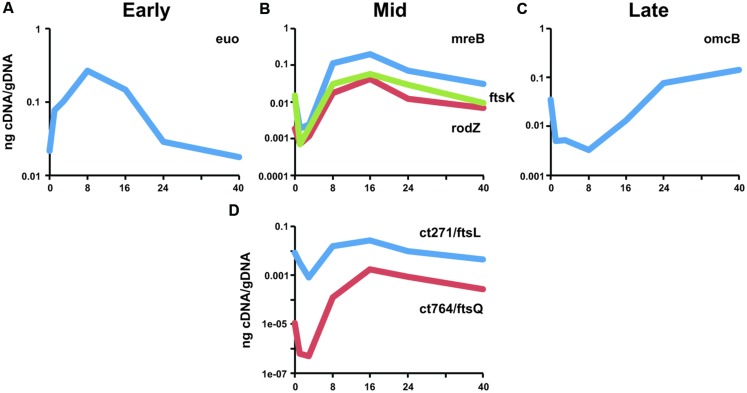
**Transcriptional analysis of *ct271/ftsL* and *ct764/ftsQ* in *Chlamydia*.** RNA was collected from infected cells at the indicated timepoints and assayed for transcripts by RT-qPCR. Transcript levels were normalized to genomic DNA (ng cDNA/gDNA). **(A)** Representative early stage (*euo*), **(B)** mid cycle division-related (*mreB, rodZ, ftsK*), and **(C)** late-stage (*omcB*) transcripts are shown for comparison. **(D)** *ct271* and *ct764* transcription are consistent with a mid-cycle profile. Data are representative of a minimum of two experiments. Standard deviations were typically less than 5% of the mean.

As cell division occurs exclusively in the RB phase of the chlamydial developmental cycle, we assayed the transcriptional profile of *ct271* and *ct764* and compared their pattern of transcription to that of previously characterized division genes (mid cycle; Ouellette et al., 2014a) and other temporally transcribed genes with functions specific to EB-to-RB (*euo*: early; Wichlan and Hatch, 1993) and RB-to-EB (*omcB*: late; Everett and Hatch, 1991) differentiation. Human epithelial cells were infected with *C. trachomatis* serovar L2 and RNA and DNA were collected from various time points over the developmental cycle. Transcripts were measured by RT-qPCR and normalized to genomic DNA since *Chlamydia* lacks a constitutively expressed gene due to its developmental cycle (Ouellette et al., 2005, 2006). The early stage gene, *euo*, is transcribed immediately after entry into a host cell as demonstrated by the positive slope between 0 and 1 h post-infection (hpi; **Figure 3A**). Conversely, the late stage gene, *omcB*, is transcribed later during the developmental cycle when RBs differentiate to EBs as demonstrated by the sharp increase between 16 and 24 hpi (**Figure 3C**). The mid cycle division genes, including *mreB*, *rodZ*, and *ftsK*, show a peak of transcription at 16 hpi (**Figure 3C**; see also Ouellette et al., 2014a). The transcription profile of *ct271* and *ct764* also peaked at 16 hpi (**Figure 3D**). Therefore, we conclude that *ct271* and *ct764* are transcribed as mid cycle genes consistent with a role in RB growth and division.

### GFP-Ct764 Localizes to the Septum of Dividing Chlamydial RBs

**FIGURE 4:**
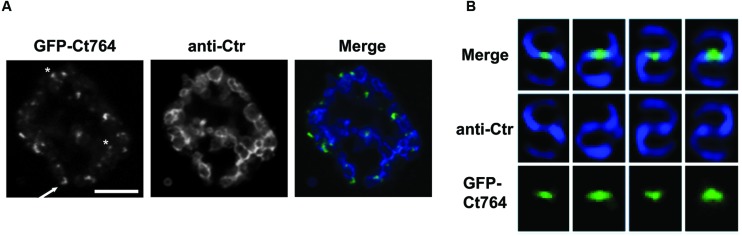
**Localization of GFP-Ct764 in *Chlamydia*.** *Chlamydia trachomatis* L2 was transformed with a tetracycline-inducible expression vector encoding GFP-Ct764. **(A)** After 18 hpi, 1 nM anhydrotetracycline (aTc) was added for 6 h. Cells were fixed and processed as described in section “Materials and Methods.” Confocal images of inclusions with multiple organisms were acquired with a 60x objective and a 2x digital zoom. The left panel shows the localization of GFP-Ct764, the middle panel an outer membrane labeling (anti-Ctr), and the right panel a merged image. Small arrows indicate crescent-like structures whereas asterisks indicate puncta. Scalebar = 5μm. **(B)** After 9 hpi, 0.06 nM aTc was added for 3 h, and cells were subsequently fixed and processed as described with the GFP-Ct764 fusion protein visualized using an anti-GFP antibody. Representative images of Ct764 localization from four pairs of dividing RBs are shown. Deconvolved images were acquired with a 100x objective.

To investigate further the function of Ct764 in *Chlamydia*, we employed recent advances in chlamydial genomics to transform *C. trachomatis* serovar L2 with an anhydrotetracycline (aTc)-inducible plasmid (Wickstrum et al., 2013; kind gift of Dr. P. S. Hefty) expressing GFP-Ct764. To date, we have not been able to successfully transform *Chlamydia* with a GFP-Ct271 construct and thus did not continue with our analysis of this protein. Human epithelial HEp2 cells were infected with plaque-purified transformants and, at 18 hpi, 1 nM aTc was added to the cultures to induce expression of the GFP-tagged construct. At varying times post-induction, cells were fixed and visualized by confocal microscopy for localization of signal after staining organisms with an antibody (anti-Ctr) recognizing the membrane of *Chlamydia* as an indicator of the bacteria. By 1 h of induction, GFP-Ct764 demonstrated clear membrane localized puncta, with occasional discrete, crescent-shaped regions, overlapping with and peripheral to sites of contact between RBs, and this pattern was consistent up to 6 h (imaged in **Figure 4A**). We also noted that the bacteria were not uniformly labeled with the GFP-Ct764, and we interpret this as reflecting that RBs divide asynchronously as seen with *E. coli* (e.g., Karimova et al., 2012). The pattern of GFP-Ct764 labeling is not distinct from what has been observed for MreB, which formed foci and bar-like structures (Kemege et al., 2015). Additionally, we previously documented FtsI and Pbp2 punctate staining using anti-peptide antibodies (Ouellette et al., 2012). However, to alleviate concerns that the foci we observed were not potential inclusion bodies or otherwise artifactual in nature, we imaged the localization of Ct764 in individual bacterial cells treated with a very low dose, 0.06 nM, of inducer at an earlier timepoint when RBs are initiating division. Here, we saw very clear localization of Ct764 at the septum of dividing chlamydiae (**Figure 4B**). It is not clear at this time whether the GFP-Ct764 is recruited at the beginning or end of the division process, thus marking points of contact between RBs, or is serving an alternative function in the division process that is distinct from the classic binary fission processes used by most bacteria.

### Over-expression of GFP-Ct764 Blocks Progression through the Developmental Cycle

**FIGURE 5:**
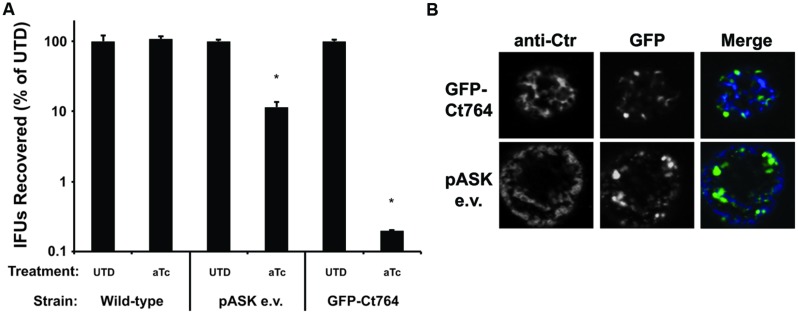
**Effect of over-expression of GFP-Ct764 on chlamydial growth and development.** Cells were infected with wild-type *C. trachomatis* L2 (Ctr L2), Ctr L2 transformed with a plasmid encoding an inducible GFP (pASK e.v.), or Ctr L2 transformed with a plasmid encoding an inducible GFP-Ct764. **(A)** At 16 hpi, infected cells were treated or not with 10 nM aTc. At 40 hpi, cells were lysed and recoverable inclusion forming units (IFUs) were quantified on fresh cell layers without aTc treatment. Data are expressed as a percentage IFUs recovered compared to the untreated (UTD) control for the given strain. Data are the average of two experiments. ^∗^indicates *p* < 0.05 by Student’s one-tailed *t*-test. See also Supplemental Figure S2. **(B)** Representative images of inclusions at 40 hpi after treatment with 10 nM aTc at 16 hpi. Note the presence of large, aberrant RBs in the GFP-Ct764-expressing bacteria compared to those expressing GFP alone (pASK e.v.). The left panels show an outer membrane labeling (anti-Ctr), the middle panels GFP fluorescence, and the right panels the merged images. The width of each panel is 10 μm.

Cell division proteins are carefully regulated and stoichiometrically balanced (Egan and Vollmer, 2015), thus disrupting this balance may impair the division process. To determine if this would also be true of Ct764 in *Chlamydia*, we compared the growth rates of wild-type *C. trachomatis* L2 to that of *C. trachomatis* L2 transformed with the tetracycline-inducible empty vector (encoding GFP alone) or the GFP-Ct764 expression vector in the presence and absence of the aTc inducer. HEp-2 cells were infected with the different strains, and, at 16 hpi, 10 nM aTc was added or not to the cultures. Bacteria were collected after 40 hpi, and infectious forms (i.e., EBs) were quantified on fresh cell layers. Wild-type chlamydiae showed no effect when treated with aTc whereas chlamydiae expressing GFP alone produced roughly 10-fold lower EBs (**Figure 5A**), likely owing to the increased metabolic burden of producing GFP. Interestingly, we observed that aTc treatment of chlamydiae expressing GFP-Ct764 led to a dramatic blockage (3 log reduction) in chlamydial development (**Figure 5A**). Expressing GFP alone did not obviously affect bacterial morphology or inclusion growth whereas over-expressing GFP-Ct764 induced abnormally large RBs and smaller inclusions (**Figure 5B** and Supplemental Figure S2). These data suggest that GFP-Ct764 can act as a dominant negative factor likely by disrupting the balance of division proteins at the septum.

### Ct764 Interacts with Division Proteins of *C. trachomatis*

**FIGURE 6:**
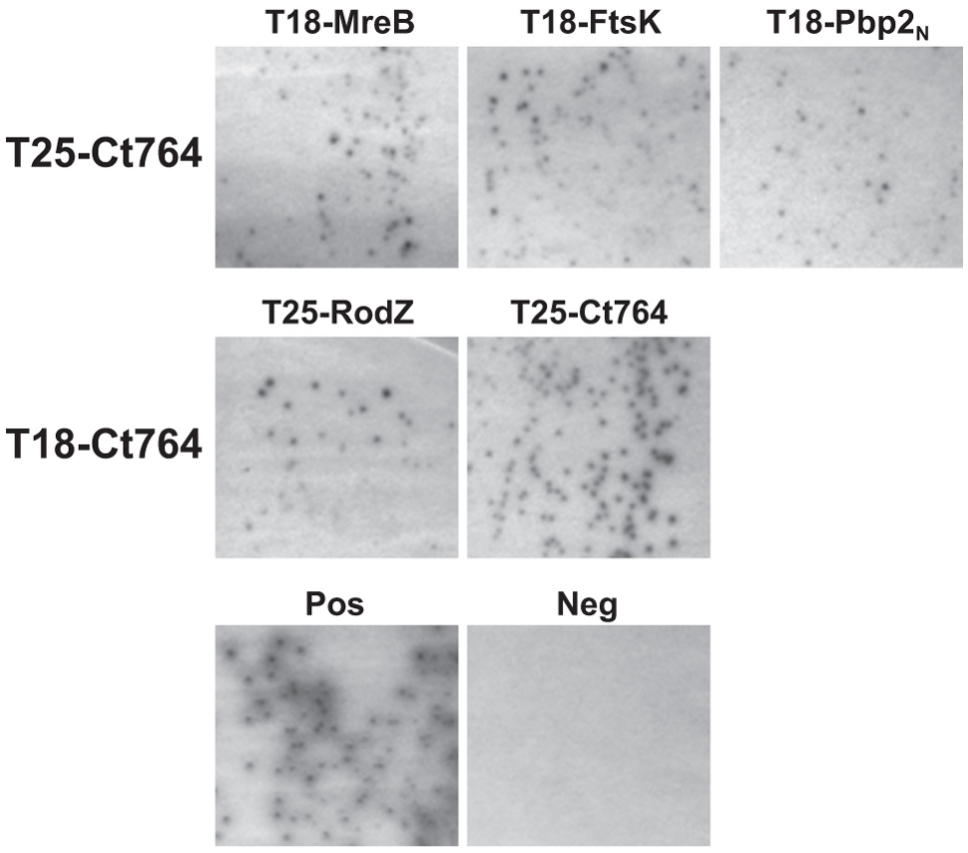
**Interactions between Ct764 and chlamydial cell division proteins.** Bacterial adenylate cyclase-based two hybrid (BACTH) assays were performed to test interactions. DHT1 *E. coli* were co-transformed with plasmids expressing the indicated fusion proteins and plated on minimal medium with maltose. Only positive interactions result in growth on maltose and dark colonies. A positive control of T25-RodZ and T18-MreB, and a negative control of T18-Ct764 and T25-Ct471 are shown. These tests were performed a minimum of two times.

One characteristic of division proteins is that they interact with other division proteins, and this trait can be exploited to identify novel division components. We employed the bacterial adenylate cyclase-based two hybrid (BACTH) system to investigate the interaction of Ct764 with known chlamydial division proteins. The BACTH system is based on the reconstitution of adenylate cyclase activity in an *E. coli* Δ*cya* mutant by bringing into close proximity two complementary fragments, T25 and T18, of the adenylate cyclase from *Bordetella pertussis*. When two proteins fused to these fragments interact, cAMP is generated, which leads to LacZ expression. A panel of constructs was made to test T25-Ct764 or T18-Ct764 versus division proteins from *C. trachomatis* fused to the T25 and T18 fragments. These plasmids were co-transformed into the DHT1 Δ*cya E. coli* and plated on X-gal containing minimal medium with maltose where blue colonies would indicate a positive interaction. Only bacteria expressing proteins that interact support growth on minimal medium and maltose. Ct764 was found to interact with MreB, RodZ, FtsK, an N-terminal fragment of Pbp2, and Ct764 itself (**Figure 6**) but not with FtsI, FtsW, or RodA. Cell division machinery is recruited in an hierarchical manner such that “late” proteins must interact with “earlier” ones (see **Figure 1**). In this context, it is particularly noteworthy that Ct764 interacted with MreB and FtsK, which are predicted to be recruited to the chlamydial division site earlier in the process. Given that FtsQ is known to interact with itself, the observed interaction of Ct764 with itself further supports its designation as a chlamydial FtsQ.

### Ct764 Interacts with *E. coli* Division Proteins but does Not Complement an ftsQ-depleted Strain

**FIGURE 7:**
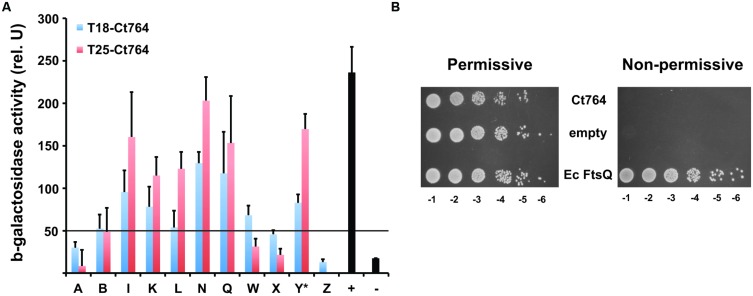
**Interactions of Ct764 with *E. coli* cell division proteins and functional complementation of an FtsQ mutant. (A)** DHT1 *E. coli* were co-transformed with T25-Ct764 (red bars) or T18-Ct764 (blue bars) and an *E. coli* cell division (Fts) protein designated by its letter (e.g., A = FtsA). Y^∗^ = YmgF. The positive control was T25-Zip vs. T18-Zip, and the negative control was T18-Ct764 vs. T25-Ct471, an unrelated membrane protein. Beta-galactosidase activity was quantified from overnight cultures as described in section “Materials and Methods.” A positive interaction is defined as having fivefold greater activity than the negative control. Here, the cutoff is approximately 50 units, indicated by the horizontal black line at this value. Data are representative of at least two experiments. **(B)** The FtsQ depletion strain, JOE417, was transformed with tetracycline-inducible plasmids expressing Ct764, *E. coli* FtsQ, or nothing (empty). Overnight cultures were subcultured and normalized for cell density, and serial 1:10 dilutions (e.g., -1 = 10^-1^) were spotted onto permissive or non-permissive agar. The same results were obtained using two different clones from different transformations of JOE417.

The ability of Ct764 to interact with chlamydial division proteins led us to ask whether Ct764 was capable of heterologous interactions with cell division proteins of *E. coli*. Here, we found that Ct764 interacted with many of the same division proteins as *E. coli* FtsQ (see Karimova et al., 2005; **Figure 7A**). Taken together, these observations support the conclusion that Ct764 is a division protein. Given the ability of Ct764 to interact with *E. coli* division proteins, we tested whether it was capable of complementing an *E. coli ftsQ* depletion strain (JOE417; Chen et al., 2002; kind gift of Dr. M. Goldberg). Here, *E. coli* growth is maintained by an arabinose inducible plasmid (pBAD33) expressing *E. coli* FtsQ in a Δ*ftsQ* genetic background. Removal of arabinose and supplementation of the medium with glucose represses the plasmid expression of FtsQ and leads to filamentation and no growth of the bacteria. We constructed tetracycline-inducible plasmids encoding either the *E. coli ftsQ* or the chlamydial *ct764* (untagged) and subsequently transformed the JOE417 strain with these plasmids or the empty vector as a negative control. Bacterial growth was then tested on agar plates containing either arabinose or glucose + aTc. As seen in **Figure 7B**, all transformed strains of JOE417 were capable of growing on permissive medium supplemented with arabinose as expected. However, in the presence of glucose and aTc, only *E. coli* expressing its native FtsQ from the aTc-inducible plasmid was able to grow. Therefore, Ct764 is not able to complement the function of FtsQ in *E. coli*.

## Discussion

Chlamydial cell division is an intriguing process due to the absence of the “essential” divisome organizer, FtsZ, as well as many other core Fts division proteins. The common ancestor of the superphylum containing *Chlamydia* is thought to have used an FtsZ-dependent mechanism (Pilhofer et al., 2008), thus loss of FtsZ is an adaption that must necessarily have been coupled to modifying an existing system to or acquiring genetic components for cell division. Neither possibility precludes conservation of key division components. Here, we identified chlamydial FtsL and FtsQ homologs, annotated as the hypothetical proteins Ct271 and Ct764, respectively, based on bioinformatics analyses. Not surprisingly, neither of these proteins shows significant sequence homology to their homologs in other organisms although we identified a conserved leucine-zipper like motif in Ct271. We have previously demonstrated that the chlamydial protein Ct009 is a RodZ homolog in spite of little sequence homology (Ouellette et al., 2014a). Thus, sequence homology is a poor indicator of functional homology. Subsequent work was designed to test whether Ct764 is a *bona fide* division protein in *Chlamydia*. We demonstrated that its transcription is consistent with other division-related genes, that it localizes to chlamydial division septa, and that it interacts with other known chlamydial division proteins. Consequently, our data support the conclusion that Ct764 should be re-designated as FtsQ.

FtsQ is a bitopic inner membrane protein containing a short cytoplasmic tail, a transmembrane domain, and a large periplasmic region containing two domains: a POTRA and C-terminal beta domain (van den Ent et al., 2008). Interestingly, the chlamydial FtsQ was unable to complement an FtsQ depletion strain of *E. coli* in spite of interacting with most of the same *E. coli* Fts proteins with which *E. coli* FtsQ interacts (Karimova et al., 2005). We suspect this may be due to the absence of an identifiable POTRA domain in the periplasmic region of chlamydial FtsQ. The POTRA domain is present in *E. coli* FtsQ and is thought to play a role in localizing FtsQ to the division plane (van den Ent et al., 2008) yet other studies suggest that divisome interactions can occur via the transmembrane domains (Karimova et al., 2009, 2012). Thus, it is possible that chlamydial FtsQ maintains the intramembrane contacts that allow it to interact with division proteins in the BACTH assay but is unable to be recruited to *E. coli* division planes by the upstream divisome components. The absence of this POTRA domain suggests that *Chlamydia* may employ a different strategy for recruiting FtsQ to its division plane.

Besides being *recruited* to the division site differently in *Chlamydia*, FtsQ may also *function* differently in this organism. There are three key observations with Ct764/FtsQ that support the likelihood that the chlamydial division process is unique compared to *E. coli*. Firstly, Ct764 clearly localized to division sites in RBs yet we were unable to resolve ring-like structures. Similarly, no ring-like structures were observed for MreB using antibodies (Kemege et al., 2015); rather, MreB labeled primarily as puncta. Secondly, Ct764 interacted with division proteins from *Chlamydia*, including MreB and Pbp2, which has not been observed, to our knowledge, in other organisms. Finally, Ct764 did not functionally complement an *E. coli ftsQ* depletion strain. The chlamydial RodZ homolog also failed to complement an *E. coli rodZ* mutant (Ouellette et al., 2014a). Thus, the chlamydial cell division proteins analyzed to date lack the functionality of their *E. coli* homologs. Clearly, more work will be required to understand the role of Ct764/FtsQ in *Chlamydia* division and whether this obligate intracellular bacterium uses a canonical or a completely unique process.

The identification of chlamydial FtsL and FtsQ homologs expands the repertoire of chlamydial cell division proteins. The division process in bacteria can be broadly categorized based on their temporal recruitment to the division plane. For example, FtsZ, FtsA, and ZipA are considered “early” division proteins and play a role in establishing the cytokinetic ring. FtsK, FtsL, FtsB, FtsQ, FtsW, FtsI, and FtsN are recruited “late” and play a role in segregating the daughter chromosomes and building and disassembling a cell wall to separate the daughter cells (Goehring and Beckwith, 2005). *Chlamydia* has annotated homologs of FtsK, FtsW, and FtsI-all “late” proteins. Thus, we reasoned it was likely that *Chlamydia* also has FtsL, FtsB, and FtsQ homologs. The presence of chlamydial FtsL and FtsQ makes it highly likely that *Chlamydia* also encodes an FtsB homolog since these three proteins together form a complex in other bacteria (Buddelmeijer and Beckwith, 2004; Villanelo et al., 2011). We have presented evidence that Ct764 is an FtsQ homolog, but more work will be required to validate Ct271 as a functional FtsL homolog. Although there is no clear FtsB homolog in *Chlamydia*, we have identified a candidate gene and are currently investigating the possibility that it may serve the role of FtsB in this organism.

Taken together, these data suggest that *Chlamydia* has preserved the majority of “late” division proteins, including the amidases necessary to separate daughter cells (Klöckner et al., 2014). The one exception is FtsN. This is not entirely surprising given the role of FtsN in binding peptidoglycan (Duncan et al., 2013) and interacting with FtsA (Busiek and Margolin, 2014). In the latter case, FtsN interacts with FtsA through its N-terminal cytosolic domain, but *Chlamydia* lacks FtsA (Stephens et al., 1998). Therefore, the lack of an FtsN homolog in *Chlamydia* can be rationalized although we cannot exclude that a divergent or functional homolog exists.

**FIGURE 8:**
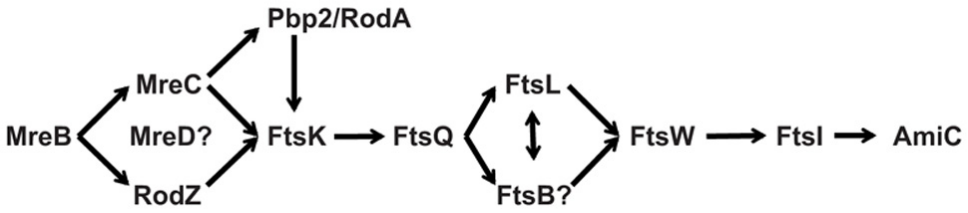
**A possible chlamydial cell division pathway.** *Chlamydia* lacks FtsZ and the early division proteins. The Mre system has been proposed to substitute for these early proteins. *Chlamydia* has maintained the late division proteins with the exception of FtsN (see text). The pathway contains known and predicted interactions.

Regardless of whether *Chlamydia* has a full complement of “late” division proteins, no homologs exist of FtsZ and the other “early” components. We previously proposed that *Chlamydia* uses MreB as a substitute for FtsZ and later identified a RodZ homolog in the bacterium (Ouellette et al., 2012, 2014a). Together with Pbp2 and RodA, *Chlamydia* also encodes an MreC homolog. The Mre system in *E. coli* and other rod-shaped bacteria consists of one additional component, MreD (Doi et al., 1988), for which there is no clear homolog in *Chlamydia*. The presence of the rod-shaped determining proteins in *Chlamydia*, which is coccoid, is clearly important, and the nearly full complement of these factors supports the likelihood that *Chlamydia* has substituted the Mre system for the “early” Fts division proteins (**Figure 8**). Further work will be required to determine the temporal order of recruitment to the division site (e.g., does FtsK recruit Ct764/FtsQ as supported by interaction data?) and whether chlamydiae use a unique division mechanism. This latter point is important as it has been assumed that *Chlamydia* divides by binary fission, however, there is no definitive experimental data supporting this assumption. Given the unique mechanism likely used for chlamydial cell division, identifying the full complement of division proteins is an important step toward unraveling this intriguing microbiological conundrum.

## Author Contributions

SO designed and performed experiments, analyzed data, and wrote the manuscript. KR and YA performed experiments and analyzed data. JC and RB analyzed data.

## Conflict of Interest Statement

The authors declare that the research was conducted in the absence of any commercial or financial relationships that could be construed as a potential conflict of interest.

## Footnotes

1 http://stdgen.northwestern.edu

2 http://ecogene.org

3 http://www.sbg.bio.ic.ac.uk/phyre2

4 http://www.ebi.ac.uk/Tools/msa/clustalo

5 http://topcons.cbr.su.se

6 www.ch.embnet.org/software/TMPRED_form.html

7 www.cbs.dtu.dk/services/SignalP

8 http://2zip.molgen.mpg.de/index.html

9 http://www.jcvi.org
