# Supplementary material for: Identification and Partial Characterization of Potential FtsL and FtsQ Homologs of Chlamydia
Source: Front Microbiol. 2015 Nov 13;6:1264. doi: 10.3389/fmicb.2015.01264 (PMC4643143; doi:10.3389/fmicb.2015.01264)
Supplement: Supplementary file 1 [file Data_Sheet_1.PDF]

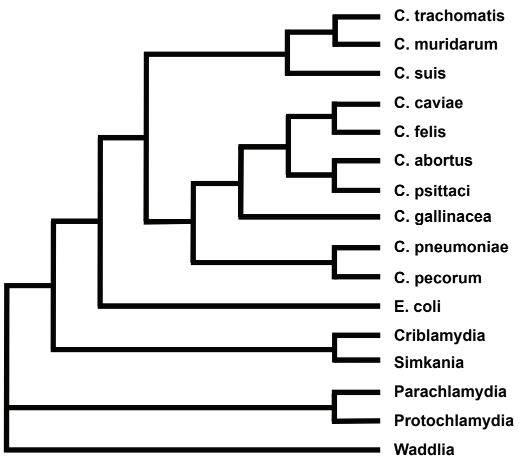


**Supplemental Figure S1**

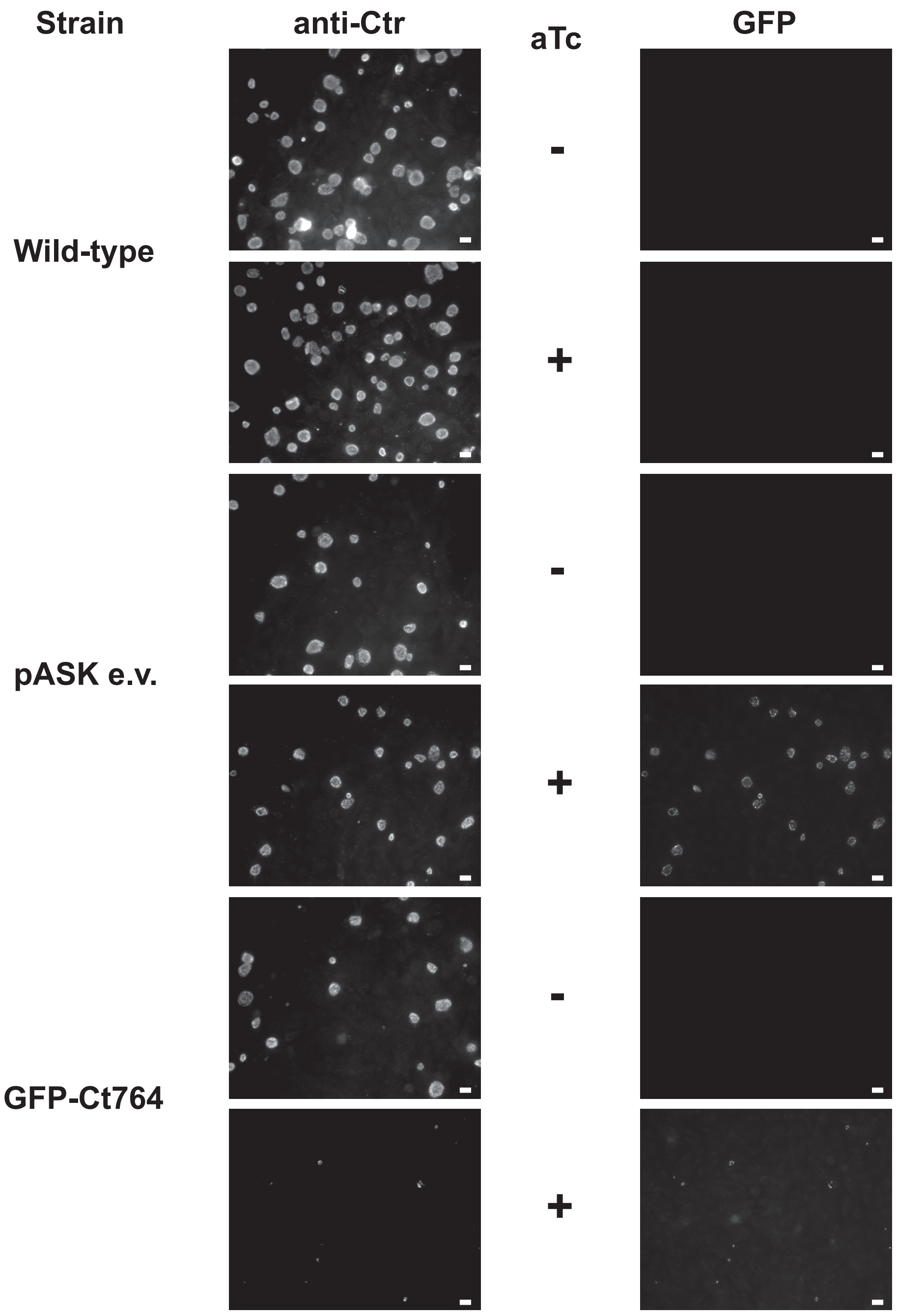


Supplemental Figure S2

Supplemental Table 1. *E.coli* strains and plasmids

| Strain | Relevant genotype [a] | | Source or Reference |
|---|---|---|---|
| JOE417* | MC4100 *araD+ ftsQE14*::*kan*/pJC10 | | (1) |
| DHT1 | $F^-$ *glnV44* (AS) *recA1 endA1 gyrA96* ($Nal^R$) *thi-1 hsdR17 spoT1 rfbD1 cya-854 ilv-691* ::Tn*10* ($Tet^R$) | | (2) |
| XL1-Blue | *recA1 endA1 gyrA96* ($Nal^R$) *thi-1 hsdR17 supE44 relA1 lac* [F' *proAB* $lacI^q$*ZΔM15* Tn*10* ($Tet^R$)] | | Stratagene (Agilent) |
| GM48 | $F^-$ *thr-1 leuB6 thiE1 lacY1 galK2* (OC) *galX* (AS) *galT22 araC14 fhuA31 tsx-78 dam-3 dcm-6 glnV44* | | CGSC |
| Construct | Relevant genotype [a] | ori | Source or Reference |
| Plasmids: | | | |
| pDONR221 | *aph att*P1-[*cat ccdB*]-*att*P2 | ColE1 | Invitrogen (Life Technologies) |
| pKT25 | *aph* $P_{lac}$::*t25* | pACYC | (3) |
| pST25 | *aadA* $P_{lac}$::*t25* | pACYC | (4) |
| pUT18C | *bla* $P_{lac}$::*t18* | ColE1 | (3) |
| pKT25-zip | *aph* $P_{lac}$::*t25-zip* | pACYC | (3) |
| pKT25-ct764 | *aph* $P_{lac}$::*t25-ct764* | pACYC | This work |
| pKT25-pbp2N | *aph* $P_{lac}$::*t25-*$pbp2_N$ | pACYC | This work |
| pKT25-ct471 | *aph* $P_{lac}$::*t25-ct471* | pACYC | (4) |
| pKT25-ct709 | *aph* $P_{lac}$::*t25-mreB* | pACYC | (5) |
| pUT18C-zip | *bla* $P_{lac}$::*t18-zip* | ColE1 | (3) |
| pUT18C-pbp2N | *bla* $P_{lac}$::*t18-*$pbp2_N$ | ColE1 | This work |
| pST25-DEST | *aadA* $P_{lac}$::*t25-att*R1-[*cat ccdB*]-*att*R2 | pACYC | (4) |

| | | | |
|---|---|---|---|
| pUT18C-DEST | *bla* $P_{lac}$::*t18-att*R1-[*cat ccdB*]-*att*R2 | ColE1 | (4) |
| pENTR764 | *aph att*L1-[*ct764*]-*att*L2 | ColE1 | PFGRC |
| pST009 | *aadA* $P_{lac}$::*t25-att*B1-[*rodZ*]-*att*B2 | pACYC | (6) |
| pST270 | *aadA* $P_{lac}$::*t25-att*B1-[*ftsI*]-*att*B2 | pACYC | (5) |
| pST682 | *aadA* $P_{lac}$::*t25-att*B1-[*pbp2*]-*att*B2 | pACYC | (5) |
| pST709 | *aadA* $P_{lac}$::*t25-att*B1-[*mreB*]-*att*B2 | pACYC | (5) |
| pST726 | *aadA* $P_{lac}$::*t25-att*B1-[*rodA*]-*att*B2 | pACYC | (6) |
| pST739 | *aadA* $P_{lac}$::*t25-att*B1-[*ftsK*]-*att*B2 | pACYC | (5) |
| pST760 | *aadA* $P_{lac}$::*t25-att*B1-[*ftsW*]-*att*B2 | pACYC | (6) |
| pST764 | *aadA* $P_{lac}$::*t25-att*B1-[*ct764*]-*att*B2 | pACYC | This work |
| pUT709 | *bla* $P_{lac}$::*t18-att*B1-[*mreB*]-*att*B2 | ColE1 | (5) |
| pUT764 | *bla* $P_{lac}$::*t18-att*B1-[*ct764*]-*att*B2 | ColE1 | This work |
| pASK-*gfp*::L2 | *bla mKate2 tetR* $P_{tet}$::*gfp*/pL2 | ColE1 | (7) |
| pASK-*gfp_ct764*::L2 | *bla mKate2 tetR* $P_{tet}$::*gfp-ct764*/pL2 | ColE1 | This work |
| pBRtet57 | *bla tetR* $P_{tet}$ | ColE1 | This work |
| *pBRtet57-ct764* | *bla tetR* $P_{tet}$::*ct764* | ColE1 | This work |
| pBRtet57-*Ec_ftsQ* | *bla tetR* $P_{tet}$::*Ec_ftsQ* | ColE1 | This work |

[a]Genotypes indicate when constructs encode in-frame the CyaA T25- or T18-domain (*t25* or *t18*) or GFP (*gfp*). The *att* recombination sites for the Gateway vectors are indicated in their position relative to the gene of interest. Note that JOE417 marked with * requires the pJC10 plasmid (pBAD33-*Ec_ftsQ*) with arabinose in the medium for survival. CGSC = Coli Genetic Stock Center. PFGRC = Pathogen Functional Genomic Resource Center.

Constructs:

The BACTH (3) and the BACTH Gateway empty vectors (4) have been described previously. Other BACTH vectors have been previously described (5,6).

pKT25-ct764 was created by digesting the pKT25 vector with *Xba*I and *Kpn*I and inserting a PCR-amplified *ct764* product (with flanking *Xba*I and *Kpn*I sites using primers 5’-TATAATCTAGAA**C**CCCCCCGCTCCCCCTCT and 5’-TCGCCCAGGTGGTACCTG**T**TTACGTTTAAGTAAAAG). pKT25-pbp2N and pUT18C-pbp2N were created by digesting the pKT25 or pUT18C empty vector with *Xba*I and *Kpn*I and inserting a PCR-amplified *pbp2* product (with flanking *Xba*I and *Kpn*I sites using primers 5’- ACTGGCTATATCTAGAC**A**AAAGAAAACGACGCACC and 5’-ATTCAGGTACCAT**T**GGGCTTCCAACAGAAGAACC) encoding the N-terminal amino acids of Pbp2 from 2-299.

All *att*L-flanked genes were introduced by recombination from pENTR clones into the corresponding BACTH-DEST plasmid using the LR protocol as described by the manufacturer (Invitrogen). For example, pENTR764 was recombined with pST25-DEST to generate pST764.

pASK-*gfp_ct764*::L2 was constructed by digesting the empty vector pASK-gfp::L2 with the restriction enzymes *Age*I and *Eag*I (flanking the *gfp* sequence) and inserting PCR-amplified *gfp* (with flanking *Age*I and *Bgl*II sites using primers 5’-ATATAACCGGT**A**TGAGTAAAGGAGAAGCACTTTTCACTGGA and 5’-ATATAAGATCT**G**AGTCCGGACTTGTATAGTTCATCCATGCCA) and *ct764* (with flanking *Bgl*II and *Eag*I sites using primers 5’-ATATAAGATCT**C**CCCCCCGCTCCCCCTCTTTTTTA and 5’-ATACCCGGCCGTTA**T**TTACGTTTAAGTAAAAGATAGTCTTCGCA).

pBRtet57 is a pBR325 derivative lacking the *cat* gene and containing the pUC57 multiple cloning site. pBRtet57-*ct764* was constructed by inserting PCR-amplified *ct764* from *C. trachomatis* L2 genomic DNA (with flanking *Sac*I and *Sal*I sites using primers 5’-ATATAGAGCTC**A**TGCCCCCCCGCTCCCCCT and 5’- CCGCCCCCCGGTCGAC**T**TA**T**TTACGTTTAAGTAAAAGATAGT) into the digested empty vector. pBRtet57-*Ec_ftsQ* was constructed by inserting PCR-amplified *ftsQ* from *E. coli* MG1665 genomic DNA (with flanking *Sac*I and *Sal*I sites using primers 5’- ATATCGAGCTC**A**TGTCGCAGGCTGCTCTGAACA and 5’-ATACGGTCGAC**T**TA**T**TGTTGTTCTGCCTGTGCCTGAT) into the digested empty vector.


1. **Chen, J. C., M. Minev, and J. Beckwith.** 2002. Analysis of ftsQ mutant alleles in *Escherichia coli*: complementation, septal localization, and recruitment of downstream cell division proteins. J. Bacteriol. **184**: 695-705.
2. **Dautin, N., G. Karimova, A. Ullmann, and D. Ladant.** 2000. Sensitive genetic screen for protease activity based on a cyclic AMP signaling cascade in *Escherichia coli*. J. Bacteriol. **182**: 7060-7066.

3. **Karimova, G., A. Ullmann, and D. Ladant.** 2001. Protein-protein interaction between *Bacillus stearothermophilus* tyrosyl-tRNA synthetase subdomains revealed by a bacterial two-hybrid system. J. Mol. Microbiol. Biotechnol. **3**: 73-82.
4. **Ouellette, S. P., E. Gauliard, Z. Antosova, and D. Ladant.** 2014. A Gateway® compatible bacterial adenylate cyclase-based two hybrid system. Env. Microbiol. Rep.
5. **Ouellette, S. P., G. Karimova, A. Subtil, and D. Ladant.** 2012. *Chlamydia* co-opts the rod-shape determining proteins MreB and Pbp2 for cell division. Mol. Microbiol. **85**: 164-178.
6. **Ouellette, S. P., K. J. Rueden, E. Gauliard, L. Persons, P. A. de Boer, and D. Ladant.** 2014. Analysis of MreB interactors in *Chlamydia* reveals a RodZ homolog but fails to detect an interaction with MraY. Front. Microbiol. **5**: 279.
7. **Wickstrum, J., L. R. Sammons, K. N. Restivo, and P. S. Hefty.** 2013. Conditional gene expression in *Chlamydia trachomatis* using the tet system. PLoS ONE **8**: e76743.

**Supplemental Figure S1.** Phylogenetic relatedness of *Chlamydiaceae* Ct764 homologs to *E. coli* FtsQ as calculated by Clustal Omega.

**Supplemental Figure S2.** Qualitative assessment of the effect of over-expression of GFP-Ct764 on chlamydial inclusion development. Cells were infected and treated as described for Figure 5 and imaged. The strain refers to wild-type *C. trachomatis* L2 (Ctr L2), Ctr L2 transformed with an empty vector expressing aTc-inducible GFP (pASK e.v.), and Ctr L2 transformed with a vector expressing an aTc-inducible GFP-Ct764. The left panel of images represents the staining of organisms with an antibody (anti-Ctr) labeling organisms. The right panel of images represents the GFP label in the corresponding samples. GFP fluorescence should only be visible in the pASK e.v. and GFP-Ct764 panels in the presence of aTc. Epifluorescent images of representative fields of view from one of two experiments is shown at 20x magnification. Scalebar = 10μm.
